# The Content of Selected Minerals, Bioactive Compounds, and the Antioxidant Properties of the Flowers and Fruit of Selected Cultivars and Wildly Growing Plants of *Sambucus nigra* L.

**DOI:** 10.3390/molecules25040876

**Published:** 2020-02-17

**Authors:** Karolina Młynarczyk, Dorota Walkowiak-Tomczak, Halina Staniek, Marcin Kidoń, Grzegorz P. Łysiak

**Affiliations:** 1 Department of Food Technology of Plant Origin, Poznan University of Life Sciences, Wojska Polskiego 28, 60-637 Poznań, Poland; mlynk@up.poznan.pl (K.M.); dorota.walkowiak@up.poznan.pl (D.W.-T.); marcin.kidon@up.poznan.pl (M.K.); 2 Institute of Human Nutrition and Dietetics, Poznan University of Life Sciences, Wojska Polskiego 28, 60-637 Poznań, Poland; halina.staniek@up.poznan.pl; 3 Department of Dendrology, Pomology and Nursery Production, Poznan University of Life Sciences, Dąbrowskiego 159, 60-594 Poznań, Poland

**Keywords:** elderberry minerals, antioxidant activity, phenolic compounds, anthocyanins, black elder, European elder

## Abstract

This study compared the mineral content and bioactive properties of flowers and fruit coming from wild elderberry plants with those of flowers and fruit harvested from elderberry cultivars grown in an orchard. Elderberry fruit and flowers were analyzed for the content of selected minerals, phenolic compounds, and anthocyanins, as well as for antioxidant activity. Mineral content was determined by the atomic absorption spectrometry method, while antioxidant activity and the content of polyphenols and anthocyanins were determined by spectrophotometric methods. Flowers were found to contain more total ash and much higher content of most of minerals, except magnesium which was present in high concentrations in fruit. Fruit showed significantly higher antioxidant activity than flowers, whereas the total phenolic content varied depending on the growing location or cultivar. The material obtained from selected cultivars growing in an orchard had higher antioxidant activity and polyphenol and anthocyanin content than the material obtained from wild plants. Fruit of the ”Haschberg” cultivar and flowers of the ”Sampo” cultivar had the best bioactive properties of the studied samples.

## 1. Introduction

Elderberry belongs to the *Adoxaceae* family, which is rather widespread in temperate regions of Europe and other continents of the northern hemisphere. Elderberry shrubs grow in the wild, but there are also elderberry cultivars, the most popular of which are ”Sampo”, ”Samyl”, ”Alleso”, ”Korsor”, and ”Haschberg”. Compared with plants growing in the wild they bear more abundant crops of larger and heavier berries [1]. Elderberry is grown mainly for its fruit, which can be used to produce juices, soft and alcoholic beverages, marmalades, or colorants. Berries are rich in various bioactive compounds, the most important of which are polyphenols, including anthocyanins, notably cyanidin-3-sambubioside-5-glucoside, cyanidin-3,5-diglucoside, cyanidin-3-sambubioside, cyanidin-3-glucoside, and cyanidin-3-rutinoside. Among other phenolic compounds present in elderberry fruit, chlorogenic acid and quercetin derivatives occur in the highest concentrations [2]. Elderberry fruit and also elderberry flowers abound with polyphenolic compounds. White or creamish white flowers do not contain anthocyanins but are rich in phenolic acids, and kaempferol, quercetin and isorhamnetin derivatives, as well as cathechin, epicathechin, and naringenin are found there [2,3,4,5]. Similar to elderberry fruit, flowers are used in the food industry to make beverages, infusions, and liqueurs. Elderberry fruit and flowers are believed to have antiviral, anti-inflammatory, and antipyretic properties, and therefore they are often used in natural medicine to produce juices or infusions to treat common cold or upper respiratory tract infections [6]. Such properties have been confirmed by some scientific studies. For example, elderberry fruit extract has been shown to block influenza virus glycoproteins and increase the expression of interleukin-8 (IL-8), interleukin-6 (IL-6), and TNF (tumor necrosis factor) [7]. Treatment with elderberry fruit extract has also been found to shorten and help ease cold symptoms [8]. Components present in elderberry have strong antioxidant properties, hence, their potential to prevent diabetes, cardiovascular, or even cancerous diseases [9,10]. Elderberry fruit also contains ascorbic acid (6 to 25 mg/100 g) [11] and elderberry seed flour is a good source of alpha-tocopherol (vitamin E) and gamma-tocopherol [12]. Elderberry fruit and flowers contain valuable mineral nutrients which include relatively large amounts of potassium, phosphorus, calcium, sodium, and magnesium, and various microelements such as iron, zinc, manganese, and copper. Unfortunately, heavy metals such as lead or cadmium, have also been identified in elderberry in some growing locations [2,5].

Polyphenols are thought to possess properties preventing many diseases, including cancer and cardiovascular diseases. In addition, minerals, especially micro elements, are very important for nutrition. Although their appropriate doses are small (less than 100 mg per day, e.g., Fe, Cu, or Zn), they have very important regulatory functions in our bodies and are responsible for the proper functioning of, for example, the blood or nervous system. Foods of plant origin are a particularly rich source of these ingredients, and all dietary recommendations indicate that they should be the basis of our diet [2,5,6]. Owing to high levels of minerals, bioactive compounds, and anthocyanin pigments, fruit and flowers of elderberry have attracted increasing interest from both consumers and the food industry [13].

However, there are only a few cultivated varieties of elderberry, and a large amount of raw material, both for domestic use and for industrial purposes and processing, is obtained from wild-growing shrubs. Often, consumers also regard fruit growing in natural conditions as more attractive and richer in nutrients. Little is known about differences in the content of bioactive compounds and minerals in elderberry fruit and flowers depending on their growing location. Therefore, this study compared total phenolic content, anthocyanins, and antioxidant activity of fruit and flowers of elderberry plants growing in the wild with the same properties of several elderberry cultivars grown in an orchard. Additionally, the level of several minerals (Ca, Mg, Fe, Cu, Zn, and Mn) in the flowers and fruit of those plants was investigated. This comparison can help with the selection of exceptionally high nutritional value, mineral content, and bioactive compounds for processing and consumption the raw material. In addition, modern elderberry cultivars are crossbred with wild plants to obtain new cultivars. Therefore, it is very important and useful to search natural habitats for varieties with above-average use value.

## 2. Results and Discussion

### 2.1. Content of Ash and Selected Minerals in Elderberry Flowers and Fruit

Ash is what remains after material has been completely burned. Ash consists mainly of macro- and microelements. The analyzed samples varied in terms of the content of ash and minerals (Table 1). There was, on average, approximately twice as much ash in elderberry flowers than in elderberry fruit. The average content of ash in flowers and fruit, in the analyzed samples, was consistent with that reported in the literature [5,14,15]. Flowers also contained more analyzed elements, namely iron, copper, zinc, and manganese. Additionally, the content of calcium was higher in flowers than in fruit (except location W3). The only exception was magnesium, which was present in considerably larger amounts in elderberry fruit than in flowers. Calcium was the dominant mineral in flowers, whereas magnesium was the most abundant mineral in fruit.

Certain regularities were identified depending on whether the samples were taken from cultivars or plants growing in the wild (Table 2). The fruit of cultivars contained more ash content than that of the fruit of wild plants, whereas there was no such difference between flowers. Kołodziej et al. (2012) noted higher concentrations of the analyzed mineral components in the flowers than in the fruit of elderberry plants growing in sixteen locations, with potassium, magnesium, and calcium being the most abundant elements in both flowers and fruit [16]. Potassium was also found to be the dominant mineral in studies that analyzed only elderberry fruit. In this study, of all the minerals measured, copper was the least abundant in both fruit and flowers, which was consistent with earlier findings [14,17].

### 2.2. Antioxidant Capacity (AC) of Elderberry Flower and Fruit Extracts

**Figure 1:**
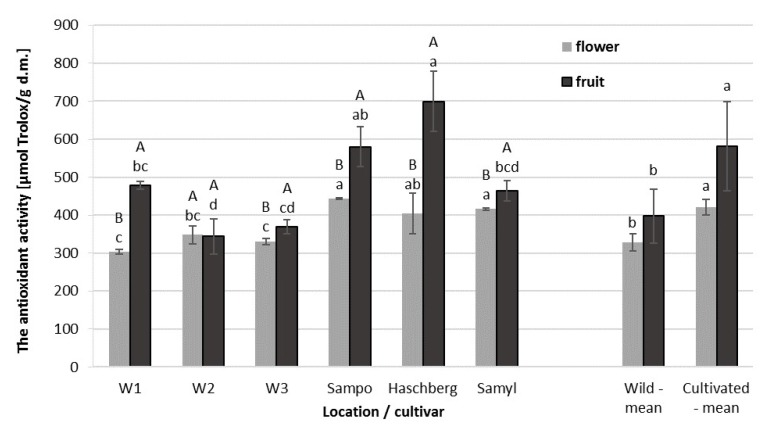
Antioxidant activity in elderberry flowers and fruit, depending on location, wild plants and cultivar. (**A**,**B**) Differences between flowers and fruit per location (wild plants) and cultivar, statistically significant at *p* < 0.05; (a,b,c,d) differences between flowers or fruit from different locations, wild plants and cultivar, statistically significant at *p* < 0.05. d.m., dry mass.

Elderberry fruit and flowers are very rich sources of antioxidants [18,19]. The elderberry fruit, analyzed in this study, had an equal or higher antioxidant capacity as compared with flowers of the same cultivars or wild plants from the same locations (Figure 1.). This stands in contrast to earlier studies by Dawidowicz et al. (2006) [20] and Kołodziej et al. (2011) [21] who used the DPPH (2,2-diphenyl-1-picrylhydrazyl) and FRAP (Ferric Reducing Antioxidant Power) methods for their measurements and found higher radical scavenging activity in elderberry flowers. In addition, fruit and flowers of the analyzed cultivars demonstrated significantly higher antioxidant activity that those harvested from wild growing plants (Table 3). As for the cultivars themselves, the fruit of ”Haschberg” had the highest antioxidant capacity. The antioxidant capacity of flowers ranged from 304 to 444 µmol Trolox/g d.m. and, similar to the case of fruit, it was higher in the flowers of cultivars than in that of wild plants.

Other authors have also analyzed the antioxidant capacity of elderberry fruit extracts. The value obtained by Silva at al. (2017) was about 430 μmol Trolox/g d.m. for fruit, which was very close to the values obtained in our study [22]. In addition, Căta et al. (2016) used the ABTS method to measure the antioxidant capacity of elderberry fruit extracts and obtained a value of approximately 350 μmol Trolox/g d.m. [23]. Only Mikulic-Petkovsek et al. (2016) found that the TEAC value for the analyzed elderberry fruit was lower (210 Trolox/g d.m.) [24]. The antioxidant capacity is significantly affected by the method and technique of analysis, as well as the growing location and conditions and individual variations in analyzed plants.

### 2.3. Total Phenolic Content (TPC) of Elderberry Flower and Fruit Extracts

**Figure 2:**
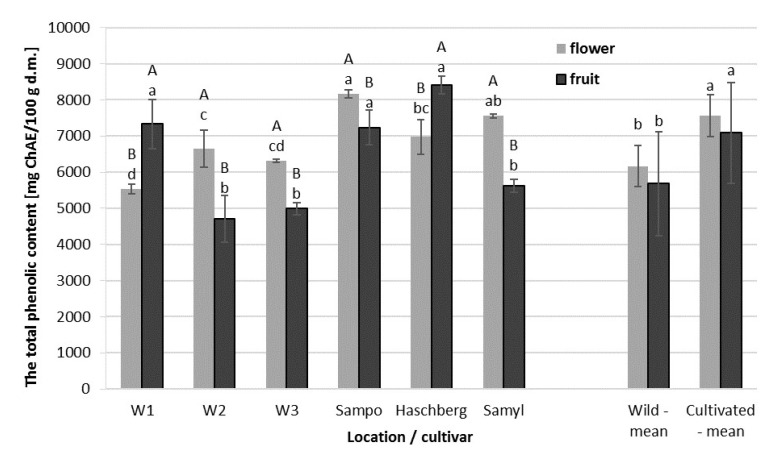
Total phenolic content in elderberry flowers and fruit, depending on location (wild plants) and cultivar. (**A**, **B**) Differences between flowers and fruit per location (wild plants) and cultivar, statistically significant at *p* < 0.05; (a,b,c,d) differences between flowers or fruit from different locations, wild plants and cultivar, statistically significant at *p* < 0.05. ChAE, chlorogenic acid equivalent.

The total phenolic content in elderberry flowers and fruit varied (Figure 2). The fruit of the ”Haschberg” cultivar and the fruit of plants growing naturally in W1 had a significantly higher content of polyphenols than the flowers from the same locations. However, this was not a general rule because flowers were richer in total polyphenols than in the fruit in the remaining samples. Nevertheless, the growing environment and the cultivar significantly affect the total phenolic content since both flowers and fruit of cultivars grown in an orchard contained significantly higher levels of polyphenols than those obtained from wild plants (Table 3). The highest total phenolic content was found in the fruit of ”Haschberg” (8405 mg/100 g d.m.), ”Sampo”, and in the fruit of wild plants from W1 (7233 to 7329 mg/100 g d.m.). Fruit collected from wild plants growing in the other two locations (W2 and W3) contained the lowest amounts of polyphenolic compounds, about 40% less than the fruit of ”Haschberg”. Total phenolic content varied less in flowers. The richest in polyphenols were the flowers of the ”Sampo” cultivar (8156 mg/100 g d.m.), followed by the flowers of ”Samyl” and, then, by those of ”Haschberg”. Additionally, the study conducted, in Denmark, by Christensen et al. (2008) suggested that polyphenol content depends on the cultivar, in agreement with our study, the total phenolic content was higher in the flowers of ”Sampo” than in those of ”Haschberg” [25].

The content of polyphenols was strongly correlated with antioxidant capacity, which is in line with the findings of numerous studies [24,26]. The correlation coefficient was 0.93 for fruit and 0.97 for flowers (Table 4). Kołodziej et al. (2011) showed that elderberry flowers contained more phenolic compounds than elderberry fruit collected from the same locations. A similar observation was made for infusions from dried elderberry flowers and dried elderberry fruit; flowers were always found to be richer in various phenolic compounds, especially flavonoids, than in the fruit [21,27].

### 2.4. Total Anthocyanin Content (TAC) in Elderberry Fruit Extracts

**Figure 3:**
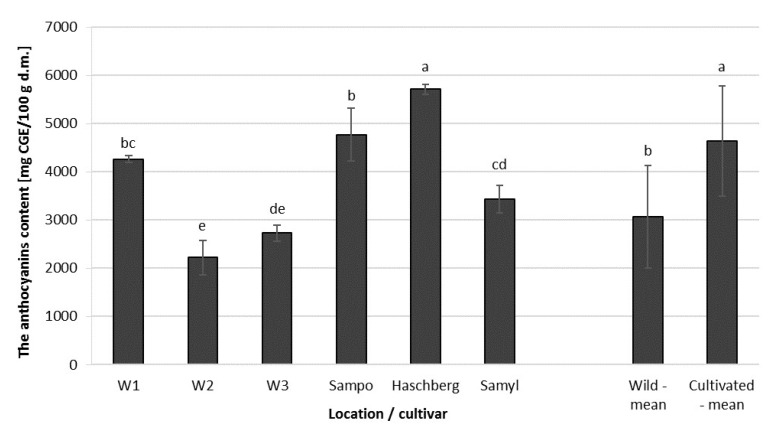
Total anthocyanin content in elderberry fruit, depending on location (wild plants) or cultivar. (a,b,c,d) Differences between average values, statistically significant at *p* < 0.05. CGE, cyanidin-3-glucoside equivalent.

Anthocyanins are pigments responsible for the dark color of elderberry fruit [28]. The content of anthocyanins in elderberry fruit was found to depend on cultivar and location (Figure 3). The largest amounts of anthocyanins were extracted from ”Haschberg” fruit. Fruit collected from wild plants growing in two locations (W2 and W3) contained less than half of that amount. ”Samyl” had the smallest amounts of anthocyanins among all the analyzed cultivars. Fruit harvested from the cultivars varied in terms of total anthocyanin content, similar to fruit of wild plants from three different locations, but fruit of the cultivars had significantly more anthocyanins than the wild fruit (Table 3).

Moreover, as could be expected [28,29], there was also a high correlation (0.98) between total anthocyanin content and antioxidant capacity and total anthocyanin content and total phenolic compounds in fruit (Table 4).

The study by Kaack et al. (1998) analyzed the level of anthocyanins in the fruit of 13 different elderberry cultivars, including ”Sampo”, ”Haschberg”, and ”Samyl’ and, according to their study, the content of anthocyanins ranged between 664 and 1816 mg/100 g of fresh mass, whereas in our study their content was lower, i.e., anthocyanins accounted for 390 to 970 mg/100 g of fresh mass. Kaack et al. also highlighted ”Sampo” and ”Samyl’ as the cultivars whose fruit was the richest in anthocyanin pigments [11], but it seems that other factors were in play that affected the level of anthocyanins in elderberry fruit. The difference could have been due to the growing location, weather conditions [30,31], or availability of nutrients because, contrary to the results obtained in this study, Kaack et al. found that ”Haschberg” fruit contained the lowest concentration of anthocyanins [11]. In addition, according to earlier work by the same author [32], the fruit of ”Haschberg” had less anthocyanins than the fruit of ”Sampo” and ”Samyl”. In 2017, Pliszka [33] recently showed that the fruit of ”Samyl” contained more anthocyanins than that of the fruit of ”Sampo”. There must be some additional factors involved here since our study shows that ”Sampo” is richer in anthocyanins than ”Samyl”. This may suggest that the influence of environmental aspects on the content of anthocyanin pigments in elderberry fruit has been, so far, underestimated.

### 2.5. Assessment of Similarities and Differences between Genotypes Using a Cluster Analysis

**Figure 4:**
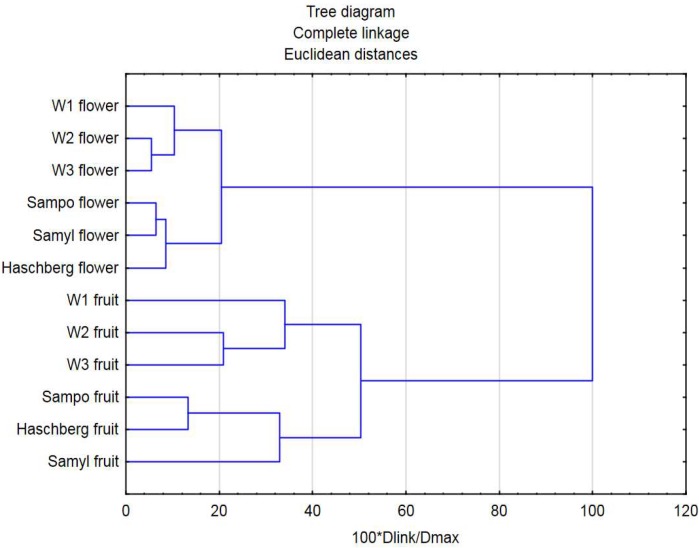
Dendrograms generated after the cluster analysis of elderberry flowers and fruit based on the content of minerals and bioactive compounds.

A complete-linkage clustering was performed to group the analyzed elderberry samples into a hierarchical cluster tree according to ash content, selected minerals, polyphenol compounds, anthocyanins, and antioxidant capacity. Figure 4 shows the results in the form of a dendrogram that divides the tested samples into clusters. The first two clusters show the division into fruit and flowers, which differ mainly in terms of the content of minerals and anthocyanins. Further grouping very clearly divides the studied samples into those coming from cultivars grown in the orchard and those originating from wild plants. The same groups were formed for both flowers and fruit, with the differences between the groups being larger for fruit than for flowers. Among the cultivars, ”Sampo” and ”Samyl” are closest to each other in terms of flower properties, whereas ”Sampo” and ”Hashberg’ are most similar to each other in terms of fruit parameters.

## 3. Materials and Methods

### 3.1. Plant Material

#### 3.1.1. Flowers

Samples were collected from their natural habitats (three different locations W1: 52°25′26.798”N, 16°54′34.437”E; W2: 52°27′34.274”N, 16°50′2.572”E; W3: 52°25′18.472”N, 16°55′47.887”E) and from an orchard (52°39′24.451”N, 16°57′10.719”E). All samples were from plants growing within a radius of about 25 km in central western Poland in order to ensure uniform climatic conditions. Three cultivars were grown in the orchard, ”Sampo”, ”Haschberg”, and ”Samyl”, and samples were taken separately from each cultivar. Elderberry flowers were collected at the full flowering stage in May 2015, by cutting the whole corymbs from wild shrubs. Corymbs were harvested in the morning and transported to a laboratory where they were cut, while discarding the thickest twigs and underdeveloped or too dry flowers.

#### 3.1.2. Fruit

Elderberry fruit was harvested when fully ripe (ripeness was judged based on color being one of the most reliable ripeness indicators [34]) from the same locations as those for flowers at the turn of August and September 2015. The whole clusters were collected and transported to a laboratory where berries were separated from pedicels and unripe, overripe and dry berries were discarded.

### 3.2. Preparation of Samples

The plant material, flowers and fruit, respectively, was divided into two parts. One part (flowers and fruit separately) was used to produce extracts. The other part was dried (flowers) or frozen at −50 °C (fruit). Flowers were dried in a laboratory drier (POL-EKO apparatus, type SLW 115 STD) using convective drying at 45 °C with an airflow speed of 2.5 m/s and variable air flow direction in the forced-air system until they were reduced to 19% to 21% of their initial mass.

### 3.3. Determination of Mineral Content by Atomic Absorption Spectrometry Method (AAS)

Dried elderberry flowers (3 g) or thawed elderberry fruit (5 g) were ashed in a muffle furnace PP330 (Nobertherm, Germany) at 550 °C. The samples were, then, burned over the burner using a nitric acid solution (Merck, Darmstadt, Germany) diluted 1:1 with deionized water. The samples were placed in a muffle furnace and reheated at 550 °C. The ash obtained in this process was weighed again and the percentage ash content in the plant material tested was calculated. The ash was then taken up in 1N nitric acid (Merck, Darmstadt, Germany) and transferred quantitatively into volumetric flasks made of polypropylene. The content of minerals such as calcium (Ca), magnesium (Mg), iron (Fe), copper (Cu), zinc (Zn), and manganese (Mn) in the obtained samples was determined by atomic absorption spectrometry with flame atomization (F-AAS) [35] using an AAS-3 spectrometer with background correction (Carl-Zeiss AAS-3 with BC, Oberkochen Germany). The studied elements were determined at the following wavelength and slit width, respectively: Ca (λ = 422.7 nm and slit = 0.50 nm), Mg (λ = 285.2 nm andvslit = 0.20 nm), Fe (λ = 248.3 nm and slit = 0.15 nm), Cu (λ = 324.8 nm and slit = 0.30 nm), Zn (λ = 213.9 nm and slit = 0.20 nm), and Mn (λ = 279.5 nm and slit = 0.20 nm). The concentrations of elements obtained in the analyzed samples were converted into content in the tested plant material and expressed in µg/g dry by simultaneous analysis mass (d.m.). The accuracy of Ca, Mg, Fe, Cu, Zn, and Mn measurements was ensured of certified reference material (Virginia Tobacco Leaves CTA-VTL-2, Poland). The average recoveries of certified levels (expressed as the average percentage of certified values) were as follows: Ca 103%, Mg 104%, Fe 97%, Cu 103%, Zn 101%, and Mn 102%.

### 3.4. Preparation of Extracts

Flower extracts were prepared by mixing 5 g of fresh flowers with 100 mL of 80% (*v*/*v*) methanol (Sigma Aldrich Co., St. Louis, MO, USA). The mixture was left for 24 h, at 4 °C, in a completely dark room and, afterwards, the extract was filtered using a vacuum pump (KNF LAB, type: N816.3KT.18, Freiburg, Germany). Extracts were prepared in triplicate and kept at −18 °C until the start of the analyses. Fruit extracts were prepared by submerging 2.5 g of fruit mash obtained using a manual homogenizer in 50 mL of 80% (*v*/*v*) methanol and then filtering it according to the procedure applied to flower extracts.

### 3.5. Measuring Antioxidant Capacity (AC) Using ABTS Radical Cation

The antioxidant capacity of extracts was determined spectrophotometrically using the ABTS (2,2’-azino-bis (3-ethylbenzothiazoline-6-sulphonic acid)) radical cation (Sigma-Aldrich Co., St. Louis, MO, USA), which was diluted with PBS (phosphate-buffered saline) buffer (pH = 7.4) until reaching an absorbance of approximately 0.730. Extracts were diluted with the same buffer to obtain four different concentrations. The resulting extract samples of 50 µL each were mixed with 5 mL of the ABTS radical solution and after 6 min of reaction at 30 °C the sample absorbance was measured at a wavelength of 734 nm, and compared with the control sample (which was mixed with PBS buffer instead of an extract sample) using a spectrophotometer (Helios Alpha, Thermo Elektron Corporation, Waltham, MA, USA). The results were expressed in µmol Trolox/1 g of dry mass [36].

### 3.6. Measurement of Total Phenolic Content (TPC)

The total phenolic content of the analyzed elderberry flower and fruit extracts was measured by the spectrophotometric method, using Folin–Ciocalteau reagent (POCH S.A., Gliwice, Poland). Distilled water was added to 100 µL of flower extract/200 µL of diluted fruit extract (2 mL of extract in 25 mL of distilled water) to obtain 1 mL of solution. Next, 5 mL of 0.2 N Folin–Ciocalteau reagent and, after, 3 min 4 mL of sodium carbonate solution (75 g/L) were added to the samples. The sample ingredients were blended, and the samples were left in a dark place for 2 h. After that time, the absorbance value of the samples was measured at a wavelength of 765 nm and compared with the reagent blank sample in a spectrophotometer (Helios Epsilon, Thermo Fisher Scientific, Waltham, MA, USA). The results were expressed in mg of chlorogenic acid/100 g of dry mass [37].

### 3.7. Measurement of Total Anthocyanin Content (TAC)

The total anthocyanin content of elderberry fruit extracts was measured spectrophotometrically, by the differential method. First, 50 µL of extract was mixed with 10 mL of buffer (pH = 1 and pH = 4.5) and the obtained samples were left in a dark place for 1 h. Afterwards, the absorbance value of the samples was measured at wavelengths of 515 nm and 700 nm and compared with a blank sample (buffer, pH = 1) using a spectrophotometer (Helios Epsilon, Thermo Fisher Scientific, Waltham, MA, USA). The results were expressed in mg of cyanidin-3-glucoside/100 g of dry mass [38,39].

### 3.8. Statistical Analysis

Statistical analysis was performed using software Statistica 13.3 (StatSoft, TIBCO Software Inc., Palo Alto, CA, USA) and MS Excel 2010 (Microsoft Corporation, Redmont, WA, USA). The results were the average of three repetitions (three extract samples) with standard deviations. The results were subjected to one-factor ANOVA and a post-hoc HSD Tukey test. The correlation between each pair of the variables, antioxidant capacity, total phenolic content, and total anthocyanin content in the samples was calculated using the Pearson correlation coefficient. In addition, a cluster analysis (Euclidean distance measure) was performed to identify groups of samples based on similarities in their measured features.

## 4. Conclusions

Elderberry flowers and fruit are, above all, a rich source of bioactive compounds with antioxidant properties, but they also contain important minerals, such as calcium or magnesium. We found that flowers and fruit had high concentrations of phenolic compounds, but fruit additionally contained high amounts of anthocyanins, and thus could show higher antioxidant capacity than flowers. Elderberry flowers, however, contained higher amounts of most of the analyzed minerals as compared with those in fruit, with no clear relationship between the origin of flowers and the mineral content. There were differences between elderberry flowers and fruit harvested from cultivars grown in an orchard and those from wild growing plants. Flowers and fruit from the cultivars were characterized by a higher content of bioactive compounds and higher antioxidant capacity than flowers and fruit from wild growing plants. Therefore, fruit and flowers of these elderberry cultivars can be a good source of highly valuable, healthy products.

## Figures and Tables

**Table 1:** The content of ash (%) and selected minerals (µg/g d.m.) in elderberry flowers and fruit, depending on the location (wild plants) and cultivar.

Raw Material	Location/Cultivar	Ash	Ca	Mg	Fe	Cu	Zn	Mn
Flowers	W1	1.5 ^d^	2673.9 ^b^	493.7 ^c^	103.0 ^b^	13.4 ^a^	39.0 ^ab^	19.7 ^e^
	W2	2.1 ^a^	3333.6 ^a^	1097.6 ^b^	87.4 ^b^	6.9 ^b^	35.2 ^ab^	60.0 ^a^
	W3	1.9 ^bc^	2925.2 ^ab^	567.2 ^c^	92.1 ^b^	13.8 ^a^	41.1 ^a^	27.9 ^d^
	Sampo	1.7 ^cd^	2868.7 ^ab^	1525.7 ^a^	54.5 ^a^	6.5 ^b^	31.8 ^b^	37.2 ^c^
	Haschberg	1.9 ^b^	2724.7 ^ab^	1555.6 ^a^	46.8 ^a^	8.0 ^b^	38.1 ^ab^	49.7 ^b^
	Samyl	1.9 ^bc^	3252.6 ^ab^	1000.0 ^b^	52.2 ^a^	13.1 ^a^	38.2 ^ab^	23.1 ^de^
Fruit	W1	0.8 ^d^	2503.2 ^b^	13555.1 ^a^	41.3 ^a^	5.2 ^b^	12.4 ^ab^	8.8 ^de^
	W2	0.8 ^cd^	2563.5 ^b^	12565.1 ^a^	35.3 ^ab^	4.4 ^b^	10.8 ^bc^	26.0 ^a^
	W3	0.6 ^e^	3033.0 ^a^	9774.3 ^b^	28.7 ^bc^	5.3 ^b^	14.0 ^a^	11.9 ^c^
	Sampo	1.0 ^ab^	662.1 ^d^	9058.1 ^b^	26.2 ^c^	4.9 ^b^	11.6 ^abc^	9.8 ^d^
	Haschberg	1.1 ^a^	1508.2 ^c^	9731.4 ^b^	28.1 ^c^	4.6 ^b^	9.5 ^c^	17.1 ^b^
	Samyl	0.9 ^bc^	1513.9 ^c^	6909.2 ^c^	25.7 ^c^	6.7 ^a^	9.8 ^bc^	8.5 ^e^

^a,b,c,d,e^ Differences between flowers or fruit from different locations, wild plants and cultivars, statistically significant at *p* < 0.05. d.m., dry mass.

**Table 2:** The average content of ash (%) and selected minerals (µg/g d.m.) in elderberry flowers and fruit, depending on raw material origin.

Raw Material	Origin	Ash	Ca	Mg	Fe	Cu	Zn	Mn
Flowers	Wild growing	1.8 ^a^	2977.6 ^a^	719.5 ^b^	94.2 ^a^	11.3 ^a^	38.4 ^a^	35.9 ^a^
	Orchard	1.8 ^a^	2948.7 ^a^	1360.4 ^a^	51.1 ^b^	9.2 ^a^	36.1 ^a^	36.7 ^a^
Fruit	Wild growing	0.7 ^b^	2699.9 ^a^	11964.8 ^a^	35.1 ^a^	5.0 ^a^	12.4 ^a^	15.6 ^a^
	Orchard	1.0 ^a^	1228.1 ^b^	8566.2 ^b^	26.6 ^b^	5.4 ^a^	10.3 ^b^	11.8 ^a^

^a,b^ Differences between average values measured in raw material, wild growing plants versus cultivars grown in orchards, statistically significant at *p* < 0.05.

**Table 3:** Average values of bioactive properties of elderberry flowers and fruit, depending on raw material origin.

Raw Material	Origin	Antioxidant Activity [µmol Trolox/g d.m.]	Total Phenolic Content [mg ChAE/100g d.m.]	Total Anthocyanin Content [mg CGE/100 g d.m.]
Flowers	Wild growing	327.7 ^b^	6164.4 ^b^	-
	Orchard	421.5 ^a^	7561.8 ^a^	-
Fruit	Wild growing	397.5 ^b^	5678.8 ^b^	3071.0 ^b^
	Orchard	581.3 ^a^	7087.3 ^a^	4638.2 ^a^

ChAE, chlorogenic acid equivalent; CGE, cyanidin-3-glucoside equivalent; ^a,b,^ differences between average values measured in raw material in wild growing plants versus cultivars grown in orchards, statistically significant at *p* < 0.05.

**Table 4:** Pearson correlation coefficients between each pair of antioxidant activity (AC) and total phenolic content (TPC) and total anthocyanin content (TAC) in elderberry flowers and fruit.

Raw Material	AC/TPC	AC/TAC	TPC/TAC
Flowers	0.97	-	-
Fruit	0.93	0.98	0.98
